# Exploring Perivascular Adipose Tissue Responses to Bioresorbable Thermoplastic Polyurethane Vascular Grafts

**DOI:** 10.34133/bmr.0372

**Published:** 2026-05-27

**Authors:** Anna-Maria Schmitt, Sabrina Rohringer, Sophie J. Specht, Selin Topcu, Christian Grasl, Heinrich Schima, Michael Prückler, Ingrid Walter, Bruno K. Podesser, Helga Bergmeister

**Affiliations:** ^1^ Center for Biomedical Research and Translational Surgery, Medical University of Vienna, Vienna, Austria.; ^2^ Austrian Cluster for Tissue Regeneration, Vienna, Austria.; ^3^ Center for Medical Physics and Biomedical Engineering, Medical University of Vienna, Vienna, Austria.; ^4^ Institute of Morphology, University of Veterinary Medicine, Vienna, Austria.

## Abstract

Substantial progress has been made in developing artificial small-diameter vascular grafts (SDVGs), yet the contribution of perivascular adipose tissue (PVAT) to vascular graft healing remains poorly understood. This study investigated the interaction of biodegradable thermoplastic polyurethane (TPU) SDVGs with PVAT using in vitro and in vivo models. Vascular and perivascular cells were cultured on TPU and analyzed by viability assays, immunofluorescence staining, scanning electron microscopy, quantitative polymerase chain reaction, and secretome and cell lysate analysis. TPU grafts were implanted into male Sprague Dawley rats for 1 week or 3 months (control = sham; *n* = 6/group) and assessed for cellularization, expression of inflammation-related genes, and adipocyte subtype markers. TPU supported viability, attachment, and phenotype preservation of in vitro-seeded cells. Immunofluorescence staining revealed increased adiponectin expression, and adipokine profiling identified 14 substantially up-regulated adipokines in TPU-seeded PVAT-derived cells. Histology showed rapid cellular coverage of grafts within 1 week of implantation, including CD34^+^ endothelial progenitor cells on the lumen. Quantitative polymerase chain reaction demonstrated compartment-specific dynamics: Inflammatory markers within the grafts changed predominantly after 3 months, whereas adjacent PVAT showed an early response with *Tnfa* induction already at 1 week. PVAT further displayed evidence of desirable immune modulation, with altered expression of innate (*Cd11c* and *Arg1*) and adaptive (*Foxp3* and *Tbet*) immune response markers, alongside partial white-to-brown adipocyte remodeling. These findings highlight PVAT’s central role—through adipokine secretion, adipocyte browning, and inflammation regulation—in vascular graft healing and underscore the importance of considering PVAT-driven adipokine dynamics in SDVG design to ensure long-term patency.

## Introduction

Advanced cardiovascular diseases often require substitution or bypass procedures to replace occluded blood vessels and restore blood flow to ischemic tissues. Autologous vessels such as the saphenous vein (SV), the internal mammary artery, or the radial artery remain the gold standard for vascular replacement, due to their favorable regenerative properties and long-term patency. However, their use is limited by restricted availability in almost a third of patients and the risks associated with invasive harvesting procedures [1].

As an alternative, synthetic vascular grafts have been developed, and clinically approved materials such as expanded polytetrafluorethylene (ePTFE) or polyethylene terephthalate are widely used. However, their performance strongly depends on the diameter of the target vessel [2]. While large-diameter conduits show acceptable performance, small-diameter (<6-mm) conduits often fail due to limited vascular ingrowth and insufficient endothelialization, which predispose them to thrombosis [3]. In addition, mechanical mismatch between the prosthesis and the native vessel further promotes the development of intimal hyperplasia [2]. These limitations necessitate intensive research on developing alternative materials with improved biomechanical properties and designs.

Thermoplastic polyurethane (TPU) has emerged as a promising candidate due to its superior elasticity, which contributes to improved compliance and biocompatibility by better matching the mechanical behavior of natural arteries [4,5]. Bioresorbable electrospun TPU small-diameter vascular grafts (SDVGs) showed favorable mechanical properties and demonstrated satisfying endothelialization as well as promising long-term survival in small animal models [6,7].

Despite these advances, the role of perivascular adipose tissue (PVAT) in the context of synthetic vascular graft healing has rarely been studied, even though it is known to have a considerable influence on vascular health. PVAT, once considered solely as structural support, is now recognized as metabolically active tissue regulating vascular tone, remodeling, and inflammation through paracrine and endocrine signaling. Functional PVAT exerts vasoprotective effects by secreting anti-inflammatory mediators such as adiponectin and hydrogen sulfide, limiting smooth muscle cell (SMC) proliferation, and promoting endothelial function [8]. By expressing endothelial nitric oxide synthase, PVAT additionally serves as a donor of nitric oxide (NO), thereby supporting vasorelaxation [9].

A clinical study by Souza et al. [10] highlighted its relevance by revealing the substantially improved patency rate of SV grafts, when harvested with the no-touch technique (95%), compared to that of conventional SV harvesting (89%) at 18 months postsurgery. This low-traumatic technique, where PVAT is preserved during SV isolation, was shown to improve vein graft patency rates by minimizing diameter mismatch and reducing the risk of occlusion in coronary circulation [11]. While primarily consisting of adipocytes, PVAT also contains macrophages, fibroblasts, mesenchymal stem cells, neurons, T cells, SMCs, and a network of vasa vasorum, supplying blood to the vessel wall [12]. This complex cellular network may allow PVAT to shape vascular healing processes by interacting with biomaterials at the graft–tissue interface and communicating closely with immune cells. Among these, macrophages are key mediators, exhibiting both pro- and anti-inflammatory responses to PVAT-derived adipokines and cytokines [9]. Depending on the PVAT-resident adipocyte subtype, PVAT was shown to modulate macrophage polarization, thereby regulating the local inflammatory environment [13].

Adipose tissue can be classified into 2 major subtypes with distinct roles in energy metabolism and thermogenesis. While white adipose tissue (WAT) stores energy by accumulating triglycerides that can be mobilized via lipolysis during catabolic states, brown adipose tissue (BAT) is known to dissipate energy to produce heat through the oxidation of fatty acids and glucose [14]. Due to the high number of mitochondria in brown adipocytes, BAT is metabolically more favorable than WAT, which is more prone to inflammation. Under pathological conditions such as obesity, diabetes, hypertension, or atherosclerosis, PVAT undergoes dysfunctional remodeling, characterized by whitening, oxidative stress, and the loss of thermogenic capacity [15,16]. Through the release of an inflammatory secretome, including mediators such as tumor necrosis factor alpha (TNFA), interleukin-6 (IL-6), monocyte chemoattractant protein-1, resistin, and leptin, white adipocytes recruit M1 macrophages and promote SMC proliferation, creating a low-grade inflammatory environment that in turn can chronically lead to insulin resistance and metabolic syndrome, followed by vascular dysfunction [17,18]. In contrast, BAT favors M2 macrophage polarization and reduces inflammation by releasing adipokines such as fibroblast growth factor-21, neuregulin-4, and vascular endothelial growth factor-A. These factors, collectively known as batokines, play an important role in improving endothelial function and mitigating vascular risk factors [19].

Unlike other adipose depots, PVAT emerges to be a heterogenic mixture of both white and brown adipocytes, with its ratio highly varying across vascular beds and their functional characteristics [20,21]. Upon different stimuli, WAT can undergo transformation into beige adipose tissue, a mechanism called browning/beiging. A study by Adachi et al. [13] revealed that injury-induced beiging of adjacent PVAT regulates its inflammatory activity and consequently affects vascular remodeling. While beige adipocytes promoted the polarization of macrophages toward the anti-inflammatory M2 phenotype, suppressing the transition from white to beige adipocytes resulted in M1 macrophage up-regulation and pathological intimal thickening. Moreover, Rohringer et al. [22] showed that PVAT inflammation in the early healing phase of SDVGs exhibited substantial differences between different vascular graft materials. In particular, ePTFE induced a stronger pro-inflammatory PVAT phenotype than autologous implants and TPU/TPU–urea (TPU/TPUU) grafts after 24 h and 1 week. In long-term implantations in a small animal model, the PVAT of TPU/TPUU implants showed a similar inflammatory and remodeling-associated gene expression pattern as autologous controls after 6 months [23].

Previous findings that showed PVAT’s active contribution to vascular remodeling as well as inflammatory regulation in native vessels suggest that it may play a similar role in the context of vascular implants [20]. Due to its proximity to the implant site, its ability to modulate immune cell behavior, and its broader role in vascular homeostasis, we hypothesized that PVAT critically affects graft integration, neointimal formation, and overall healing. Previous studies showed that different graft materials affect PVAT inflammation [22,23], but the influence of vascular graft materials on the plasticity and phenotype of PVAT has not yet been investigated. Therefore, the current study aimed to investigate the healing of TPU-based SDVGs with a particular focus on PVAT response and biomaterial-induced adipocyte subtype transition. In summary, investigating the response of PVAT and its cellular components to synthetic blood vessel prostheses could provide essential insights into how the local tissue environment affects SDVG healing and long-term patency.

## Materials and Methods

### Graft fabrication

**Fig. 1.**
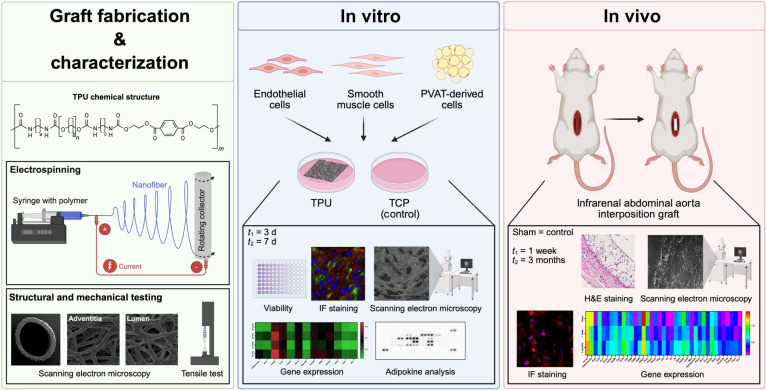
Schematic overview of the study design and analyses. Following graft fabrication and characterization, performance was assessed in vitro and in vivo through implantation in a small animal model. TPU, thermoplastic polyurethane; TCP, tissue culture plate.

The synthesis of the TPU used in this study was performed by Covestro (Germany) according to a protocol described in a previous study [6]. Poly(tetrahydrofuran) (3.65 g, 3.65 mmol) was dried at 90 °C in vacuo (5 mbar) under magnetic stirring before hexamethylene diisocyanate (1.23 g, 7.30 mmol) was diluted in 5 ml of dry dimethylformamide (DMF) and added to poly(tetrahydrofuran) in an argon atmosphere. After adding 0.15 ml of tin(II) 2-ethyl hexanoate to the reaction flask, the reaction mixture was incubated for 2 h at 90 °C under stirring. Finally, bis(2-hydroxyethyl) terephthalate (0.98 g, 3.65 mmol) was dissolved in 5 ml of DMF and added for another hour of stirring at 90 °C. After diluting the product in 70 ml of DMF, the polymer was precipitated using 1.5 l of methanol and afterward filtered, reprecipitated, and dried under vacuum. Subsequently, the degradable TPU (chemical structure shown in Fig. 1) was dissolved at 8 wt% in a mixture of 15% 1,1,1,3,3,3-hexafluoro-2-propanol and 85% chloroform. Electrospinning of the grafts was carried out using an 11-cm needle-to-target distance, a flow rate of 2.6 ml/h, an applied voltage of 11 kV, and a mandrel rotation speed of 250 rpm. Conduits with a length of 2 cm, an inner diameter of 1.5 mm, and a wall thickness of 140 ± 10 μm were sterilized with ethylene oxide and used for further testing.

### Graft characterization

Fiber morphology was determined by scanning electron microscopy (SEM) using a Zeiss EVO 10 microscope (Zeiss, Germany). TPU specimens were placed on sample holders, sputter-coated with 40-nm gold and scanned at 5,000× magnification to determine polymer fiber diameters (*n* = 20 measurements on 3 grafts at the inner and the outer side) using the length measurement tool in the ImageJ software (National Institutes of Health, USA), with the scale bar serving as a reference.

The mechanical properties of the vascular grafts were assessed by hoop tensile testing according to a previously established protocol [24]. Briefly, 11 ring-shaped graft segments (length: 2 mm) from 4 individual grafts total were mounted onto 2 aligned steel pins (diameter: 0.6 mm) in a tensile testing system (ElectroForce LM1 TestBench, TA Instruments, USA). The pins were displaced at a constant rate of 10 mm·min^−1^ to apply circumferential stress until graft failure.

Compliance measurements were conducted over a physiological pressure range of 80 to 120 mmHg and expressed as the percentage change in diameter per 100 mmHg. All experiments were performed in isotonic saline at 37 °C. Suture retention strength (*n* = 4) was measured on grafts approximately 20 mm in length, with one end clamped to an arm of the Messphysik Beta 10-2.5 tensile testing machine (Messphysik Materials Testing, Austria). On the free end of the sample, a 7.0 polypropylene suture loop (Ethicon, USA) was placed 2 mm from the edge and clamped to the other arm of the testing machine, which moved at a constant speed of 120 mm/min until failure. To calculate the gravimetric porosity of the electrospun grafts (*n* = 5), the total prosthesis volume was determined by measuring the graft wall thickness at 0.15-mm intervals using an optical micrometer (optoControl 2600, Micro-Epsilon Messtechnik, Germany), while the graft mass was recorded with an analytical balance (Micro MC 210P, Sartorius, Germany) [25].

### Cell culture

Rat aortic endothelial cells (ECs) as well as rat aortic SMCs were purchased from Innoprot (Spain). PVAT cells were isolated from rat aortas and subjected to outgrowth cultures. All cells were cultured in Endothelial Growth Medium-2 (Lonza, Switzerland) supplemented with additional 10% fetal bovine serum (Thermo Fisher Scientific, Austria) and 1% penicillin/streptomycin (Thermo Fisher Scientific, Austria) under standard cell culture conditions (37 °C, in 5% CO_2_ and 95% humidity). The medium was changed every 2 to 3 d, and as soon as cells reached 80% confluency, cells were split. Cells between passages p2 and p6 were used for the experiments.

### In vitro biocompatibility

To test the biocompatibility of the described grafts, ECs, SMCs, and PVAT cells were seeded on 2 × 2 mm TPU graft specimens with tissue culture plates (TCPs) used as control. Specimens were placed into a 96-well plate, and 1.5 × 10^4^ cells of each cell type were seeded and cultured under standard cell culture conditions for up to 7 d (*n* = 3). On days 1 and 3, a dimethyl thiazolyl diphenyl tetrazolium salt (MTT) assay was performed according to the ISO 10993-5 standard. After incubation in MTT solution (1 mg/ml; Sigma-Aldrich, Germany) for 4 h, the solution was discarded and dimethyl sulfoxide was added for another 10-min incubation on a plate shaker. The absorbance in 100 μl was measured at 590 nm (ref. 650 nm) with a Spark microplate reader (Tecan Group, Switzerland).

### Scanning electron microscopy

To investigate the surface of the TPU grafts as well as the morphology of attached cells, samples were subjected to SEM. In vitro-seeded specimens were taken at days 3 and 7, while implanted grafts were sampled after 1 week or 3 months. Samples were fixed in 2.5% glutaraldehyde (Sigma-Aldrich, Germany) overnight at 4 °C, followed by incubation in an increasing ethanol series and hexamethyldisilazane (Sigma-Aldrich, Germany) for final dehydration. After coating with 40-nm gold using a Q150R ES sputter coater (Quantum Design, Germany), samples were analyzed by SEM.

### Immunocytochemistry

Immunofluorescence (IF) staining of cells seeded onto TPU grafts was performed after 3 and 7 d with glass coverslips serving as controls. After 3 washing steps using phosphate-buffered saline (1× PBS; Thermo Fisher Scientific, Austria), samples were fixed in 4% paraformaldehyde (Sigma-Aldrich, Germany) overnight at 4 °C. The next day, samples were washed with 1× PBS and incubated in PBS/1% bovine serum albumin (BSA; Sigma-Aldrich, Germany) at room temperature for 30 min. Samples were incubated in primary antibody diluted in PBS/1% BSA for 1 h at room temperature. To visualize the cytoskeleton in SMCs, Phalloidin-iFluor 555 (ab176576, Abcam, United Kingdom) was used.

After washing thrice with PBS/1% BSA, samples were incubated for another hour with secondary antibodies diluted in PBS/1% BSA. Subsequently, samples were again washed with PBS/1% BSA, placed on microscopy slides, and embedded in 4′,6-diamidine-2-phenylindole (DAPI; Thermo Fisher Scientific, Austria) containing a mounting medium (Agilent Technologies, Austria). Until imaging with a Zeiss LSM900 confocal microscope (Zeiss, Germany), samples were stored at 4 °C in the dark. Table S1 shows a list of all antibodies used.

### Gene expression analysis

To analyze the expression of various graft-healing-related as well as inflammation-related cell markers and to examine adipocyte subtype composition in PVAT, quantitative polymerase chain reaction (qPCR) was performed with in vitro and in vivo samples. RNA was isolated using a FavorPrep Tissue Total RNA purification kit (Favorgen, Austria) according to the manufacturer’s instructions. After assessing RNA yield and quality using a Spark plate reader, samples with a 260/280 nm ratio higher than 2 were reverse-transcribed to complementary DNA using a reverse transcription kit (Qiagen, Germany). For each reaction, complementary DNA was mixed with the appropriate primers (Microsynth, Austria) and SYBR Green PCR Master Mix (Applied Biosystems, USA) and qPCR was subsequently run using a CFX Opus 96 thermocycler (Bio-Rad, Austria). Further information about the qPCR run protocol and primer sequences are described in Tables S2 and S3.

### Adipokine array

The Proteome Profiler Rat XL Adipokine Array kit (R&D Systems, Germany) was used to analyze the levels of 30 different adipokines expressed and secreted PVAT-derived cells in response to the graft material. Therefore, cells were seeded onto TPU specimens (1 × 1 cm) and cultured under standard cell culture conditions for 5 d, with TCP-seeded cells serving as controls. Cell lysates as well as supernatants were processed according to the manufacturer’s instructions, mixed with a detection antibody cocktail and subsequently incubated on array membranes overnight. Following streptavidin conjugation, signals were obtained by exposing membranes to x-ray film using a ChemiDoc imaging system (Bio-Rad, Austria). The intensity of protein spots on membranes was quantified with the ImageJ software by measuring the integrated density of each spot. Subsequent data processing was performed using RStudio (version 2026.01.0; R Foundation for Statistical Computing, Austria). Raw intensity values were first background-corrected using the mean intensity of negative control spots and afterward normalized to the mean intensity of the reference spots on each membrane individually. For supernatant samples, medium control measurements were used to calculate a limit of detection (LOD; defined as mean + 3 × standard deviation [SD] of the medium control) to exclude false-positive signals. Proteins with values below the LOD were not further investigated. After computing log_2_ fold changes and negative log_10_-transformed *P* values using RStudio, results were visualized as bubble plots using the ggplot2 package. Proteins with −log_10_(*P* value) higher than 1.3 were regarded as substantially regulated and clustered into protein–protein interaction networks based on the STRING database using Cytoscape [26]. The interaction confidence threshold is defined as the likelihood of a given interaction between 2 proteins to be true, based on available evidence, and is a property of the interaction rather than of the individual proteins. A high confidence threshold (0.9) was applied, equivalent to an estimated false-positive rate of 10%. The role of proteins within the resulting networks and their involvement in particular biological processes were identified by conducting an overrepresentation analysis based on the Gene Ontology (GO) database for biological processes, molecular functions, and cellular components [27,28].

### Animal model

The animal experiments were reviewed and approved by the Institutional Ethics Committee and received authorization from the Federal Ministry of Science and Research (GZ: BMWFW-2024-0.259.878). All procedures involving animals were conducted in compliance with the Good Scientific Practice Guidelines of the Medical University of Vienna. Anesthetic and analgesic protocols were applied as previously described [24]. The animals were anesthetized using 100 mg/kg ketamine and 5 mg/kg xylazine and orally intubated for volume-controlled ventilation with 40% oxygen and 2% isoflurane. After dissection of the aorta, electrospun TPU grafts (length = 2 cm, inner diameter = 1.5 mm) were flushed with physiological saline solution containing 5 IU/ml heparin before being implanted into the infrarenal aorta of male Sprague Dawley rats (500 to 600 g; Charles River, Germany) for 1 week and 3 months (*n* = 6 per group). Using a surgical microscope (Zeiss, Germany), end-to-end anastomosis was performed with nonabsorbable sutures (Monosof 10/0, Covidien, USA). The animals received 3 mg/kg piritramide (Janssen-Cilag Pharma, Austria) and 5 mg of carprofen (Rimadyl, Pfizer, Austria) as a perioperative analgetic regimen. After functional control of the anastomoses, both the abdominal wall and skin were closed using Vicryl 4/0 (Ethicon, USA). At the study endpoint, animals were anesthetized similarly to the implantation procedure and retrieved grafts were cut into samples for subsequent histology, SEM, and gene expression analysis. Sham animals (*n* = 6) were anesthetized similarly to the experimental group. The aorta was prepared as for the implantation procedure, and the animals were kept in anesthesia for the usual duration of graft implantation surgery (1.5 h) before the abdominal wall was closed. During or after the surgery, anticoagulation therapy was not administered in either the experimental group or the sham group. All animals received piritramide through the drinking water during the first days postsurgery.

### Hematological analysis

At the endpoint of the experiments, whole blood was collected from anesthetized animals via cardiac puncture and transferred into tubes containing either ethylenediaminetetraacetic acid anticoagulant for complete blood count/differential count analysis or into lithium heparin tubes for subsequent clinical chemistry analysis. The complete blood count and clinical biochemistry panels were performed using ADVIA 2120i Multispecies (Siemens Healthcare Diagnostics, Austria) and Cobas c311 (Roche Diagnostics, Austria), respectively. A summary of analyzer methods is provided in Table S4.

### Histology and immunohistochemistry

Histological analyses of explanted grafts were performed as previously described [23]. Samples were incubated in 4% paraformaldehyde overnight and afterward stored in 70% ethanol. After dehydrating the samples in an ascending ethanol series, a Tissue-Tek VIP 6 automated embedding device (Sakura Finetek, USA) was used for paraffin embedding; 3-μm sections were stained using hematoxylin and eosin to visualize overall tissue organization and cell structure.

In addition, longitudinal sections of explanted grafts were immunohistochemically analyzed for transanastomotic ingrowth of ECs and luminal attachment of endothelial progenitor cells. Therefore, the luminal side was stained with DAPI and with an antibody against cluster of differentiation 34 (CD34; ab81289, Abcam, United Kingdom) using the same staining procedure as for immunocytochemistry.

### Statistical analysis

All in vitro experiments were conducted with a minimum of 3 biological replicates, using pooled donor cell batches at varying passages to enhance experimental robustness. For statistical analysis, the Prism 10 software (GraphPad, USA) was used and data were tested for normality of distribution by performing a Shapiro–Wilk test. If all groups were consistent with a Gaussian distribution (*P* > 0.05), they were further evaluated for equality of variances via the Brown–Forsythe test. In case of variance equality (*P* > 0.05), an ordinary one-factorial analysis of variance (ANOVA) with a Tukey posttest was performed to analyze differences between in vivo groups. If variances were unequal (*P* ≤ 0.05), Welch’s ANOVA with a Dunnett T3 post hoc test was used. In case that any group failed the normality test (*P* ≤ 0.05), the Kruskal–Wallis test with Dunn’s multiple comparisons was conducted. For in vitro data, paired *t* tests (or Welch’s correction if variances were unequal) were performed in case of normality. If data were not normally distributed, nonparametric alternatives were considered but not used due to power limitations based on a low replicate number. *P* values < 0.05 were considered statistically substantial, and significances are represented as asterisks (***P ≤ 0.001; **P ≤ 0.01; *P ≤ 0.05). All data are presented as mean ± SD.

## Results

### Electrospun TPU grafts show consistent fiber morphology and mechanical strength

Electrospun TPU grafts were mechanically analyzed to evaluate their physical properties. A random fiber alignment was observed at the inner and the outer side, as shown by SEM images (Fig. S1A). Comparing the mean fiber diameter between the inner and outer sides of the graft, a small difference was observed. While it was calculated as 2.60 ± 0.85 μm at the luminal side, fibers on the outer side reached a mean diameter of 2.27 ± 0.84 μm, as shown in Fig. S1B. Grafts showed a mean compliance of 4.8% ± 0.5%/100 mmHg and a mean ultimate tensile force of 1.49 ± 0.12 N (Fig. S1C and D). Figure S1E shows the suture retention of electrospun prostheses with a mean retention of 118.25 ± 18 gram-force. The gravimetric porosity of the electrospun TPU grafts (*n* = 5) was 52.4% ± 2.3% (Fig. S1F).

### TPU supports cell adhesion, proliferation, and anti-inflammatory signaling in vitro

**Fig. 2.**
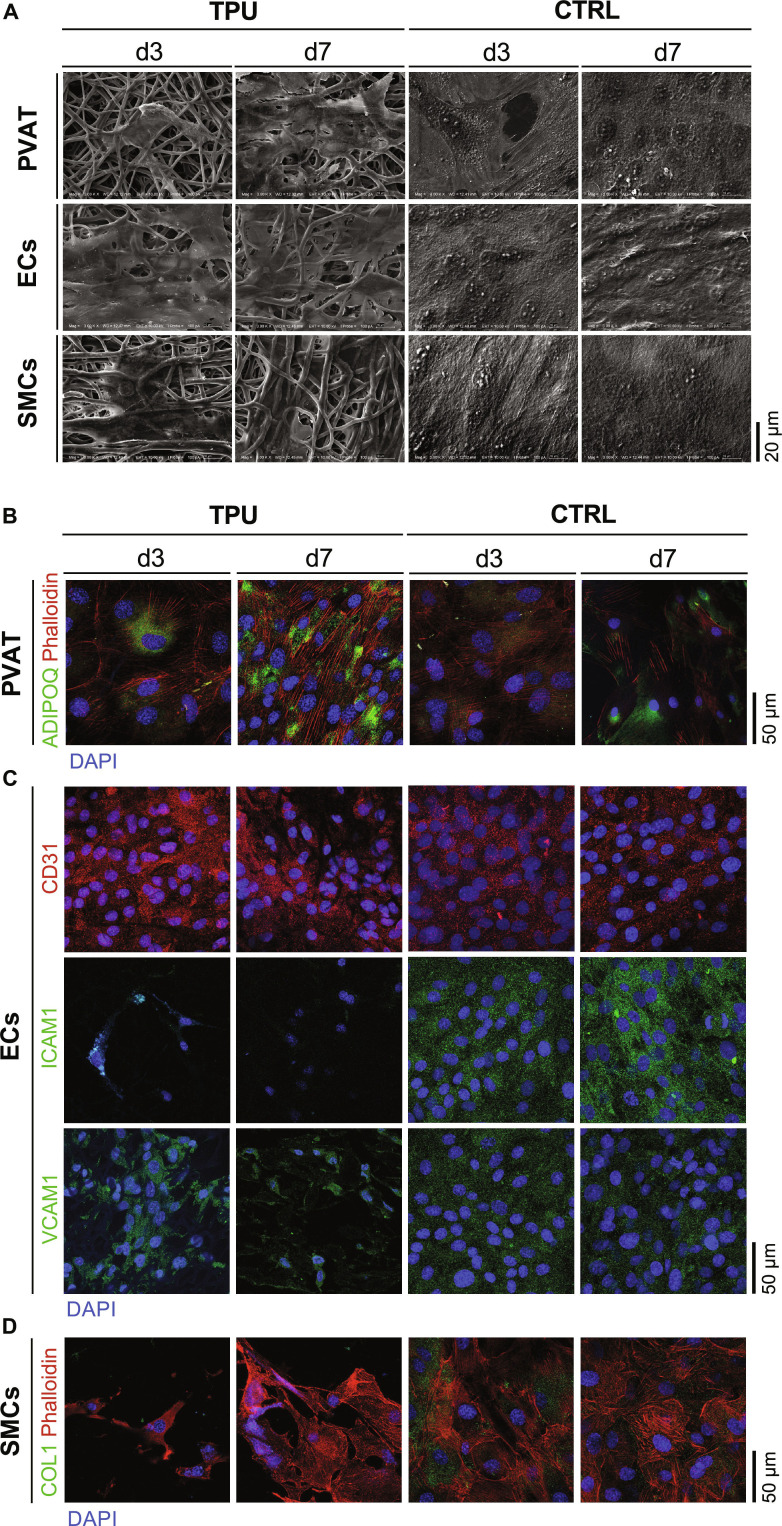
In vitro-seeded cells on thermoplastic polyurethane (TPU) vs. CTRL. (A) Scanning electron microscopy (SEM) analysis of in vitro-seeded cells on TPU specimens vs. glass (CTRL). Scale bar = 20 μm. (B to D) Immunofluorescence (IF) staining showing the expression of cell-specific markers. PVAT, perivascular adipose tissue (PVAT)-derived cells; ECs, endothelial cells; SMCs, smooth muscle cells. 4′,6-Diamidine-2-phenylindole (DAPI) = nuclei. Scale bar = 50 μm.

To assess the cytocompatibility of electrospun TPU grafts with different rat-derived vascular cell types, ECs, SMCs, and PVAT-derived cells were seeded onto TPU scaffolds in vitro. All cell types adhered to the material and proliferated until day 3 (Fig. S2). ECs displayed the lowest proliferation rate, whereas SMCs showed the highest yet nonsignificant increase in cell number between day 1 and day 3. PVAT-derived cells proliferated at a rate similar to that of ECs. Successful attachment was further confirmed by SEM performed on days 3 and 7 with glass coverslips serving as controls (Fig. 2A). On control surfaces, all cell types formed a confluent layer by day 3, whereas on TPU grafts, subconfluent layers were reached at day 7.

IF staining demonstrated that all investigated cell types showed increased anti-inflammatory and healing-associated marker expression on TPU compared to CTRL (Fig. 2B to D). In PVAT-derived cells, anti-inflammatory adiponectin (ADIPOQ) expression was higher on TPU compared to that on controls on day 3, with a further increase by day 7 (Fig. 2B). In addition, the proportion of adiponectin-positive cells increased from partial expression at day 3 to nearly all cells positive at day 7. Although adiponectin signal also increased in some cells on the control surfaces, the overall number of adiponectin-positive cells declined over time. ECs were shown to express cluster of differentiation 31 (CD31) consistently at all timepoints under both conditions, with slightly increased signals on TPU (Fig. 2C). In contrast, the expression of the pro-inflammatory adhesion molecules intercellular adhesion molecule 1 (ICAM1) and vascular cell adhesion molecule 1 (VCAM1) decreased from day 3 to day 7 on TPU, while both markers increased over time in the control group. Staining for collagen type I (COL1), a marker of the synthetic anticontractile SMC phenotype, revealed minimal expression on TPU, while cells grown on glass controls exhibited strong signals at both timepoints (Fig. 2D).

### TPU promotes adipocyte identity, transient endothelial activation, and SMC contractility

**Fig. 3.**
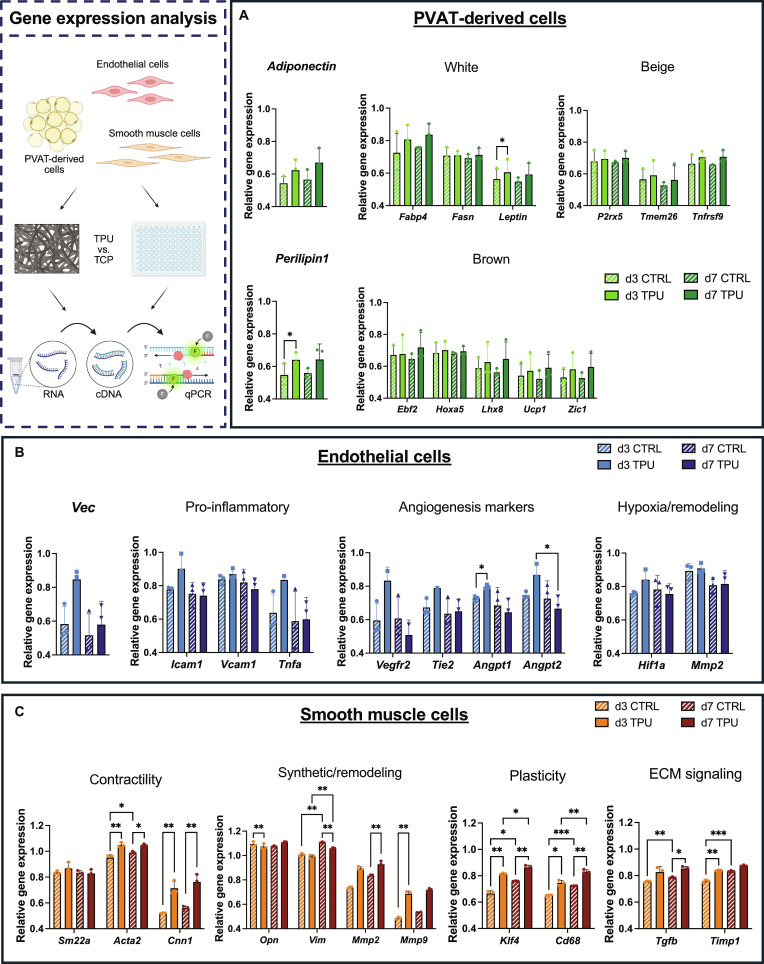
Gene expression analysis of cells exposed to thermoplastic polyurethane (TPU) vs. CTRL in vitro. (A) Perivascular adipose tissue (PVAT)-derived cells, (B) endothelial cells, and (C) smooth muscle cells. Gene expression is displayed relative to the expression of Gapdh as a housekeeping gene and presented as mean ± SD. ***P ≤ 0.001; **P ≤ 0.01; *P ≤ 0.05.

Gene expression analysis was performed on all in vitro-seeded cells. To evaluate the influence of TPU, expression profiles were compared to those of cells cultured on TCPs. The expression of markers for white, beige, and brown adipocytes was analyzed in PVAT-derived cells to additionally determine whether the material influences adipocyte subtype transitions. Adiponectin, an anti-inflammatory adipokine, showed a trend toward higher expression (Fig. 3A), and perilipin-1 (*Plin1*) as a marker for adipocyte identity showed substantially higher levels in PVAT-derived cells on TPU than in those on TCPs. While almost all markers, independently of white/beige/brown association, showed higher levels in TPU-seeded cells, *leptin* was the only subtype-specific marker (white) substantially elevated. No considerable changes or differences between the groups were observed for beige and brown adipocyte markers.

In contrast, distinct trends emerged in ECs (Fig. 2B). While the EC-identity marker vascular endothelial cadherin (*Vec*) showed a remarkable elevation in TPU-seeded cells at day 3, its levels decreased until day 7, almost reaching control levels. Initially increased expression of inflammatory markers (*Icam1*, *Vcam1*, and tumor necrosis factor alpha [*Tnfa*]) in the TPU group stabilized until day 7 on TPU, mirroring the IF results. This initial up-regulation was also observed for angiogenesis-related genes: angiopoietin-1 (*Angpt1*) and angiopoietin-2 (*Angpt2*) were strongly up-regulated at day 3 but declined again below control levels by day 7, while no comparable changes were observed in the control group. Despite an initial up-regulation of hypoxia-inducible factor 1 alpha (*Hif1a*), no remarkable changes were observed in hypoxia/remodeling markers.

The gene expression analysis of SMCs cultured on TPU versus TCPs revealed highly substantial changes over time (Fig. 3C) in almost all markers. While markers associated with the contractile phenotype (alpha-actin 2 [*Acta2*] and calponin 1 [*Cnn1*]) showed substantial elevation in TPU-seeded cells compared to controls, synthetic and remodeling phenotype markers (osteopontin [*Opn*]) and vimentin [*Vim*]) were expressed at lower levels in the TPU group while rising in controls over time. Similarly to matrix metallopeptidase 2 (*Mmp2*) and metallopeptidase 9 (*Mmp9*), markers for plasticity (Krüppel-like factor 4 [*Klf4*] and cluster for differentiation 68 [*CD68*]) were up-regulated during cultivation on both substrates but reached substantially higher levels on TPU. Transforming growth factor beta (*Tgfb*) and tissue inhibitor of metallopeptidase 1 (*Timp1*) levels also exhibited a remarkable increase, especially within the control group itself.

### TPU alters the adipokine profile in PVAT-derived cells

**Fig. 4.**
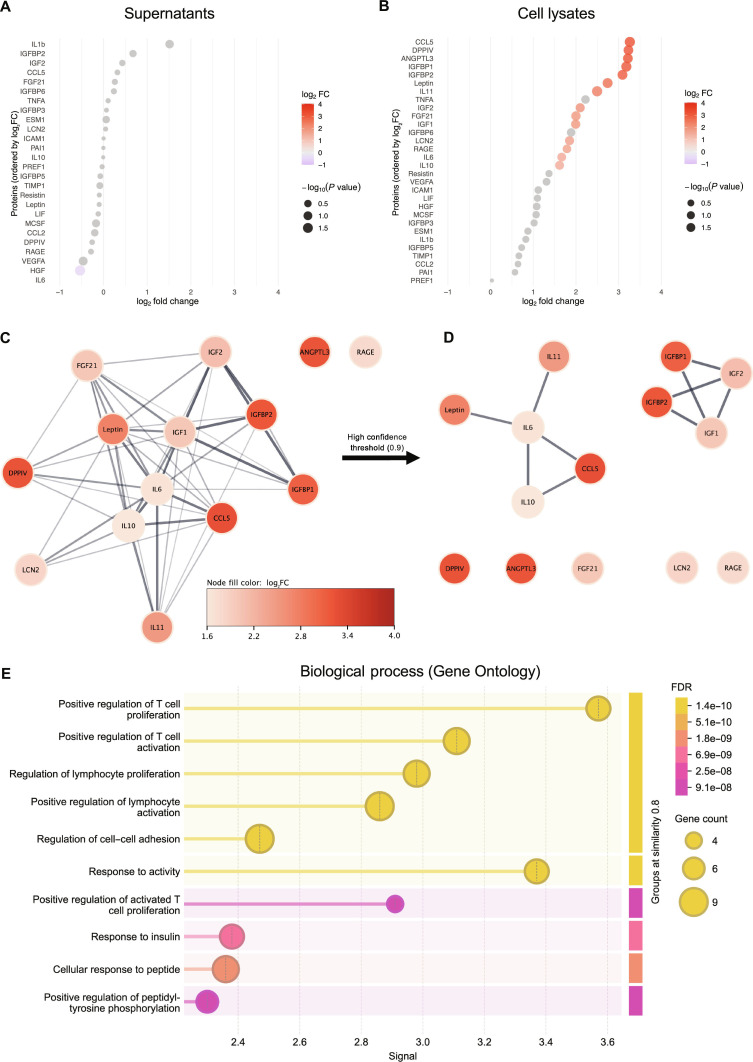
Adipokines detected in the (A) secretome and (B) cell lysates of perivascular adipose tissue (PVAT)-derived cells seeded onto thermoplastic polyurethane (TPU) in vitro displayed in bubble plots. (C) STRING network of substantially up-regulated adipokines in cell lysates, which was further refined and displayed a strong connection within (D) 2 main clusters. (E) Gene Ontology overrepresentation analysis of these adipokines, filtered by biological process.

In addition to changes in gene expression level, we analyzed adipokines in the secretomes and cell lysates from PVAT-derived cells seeded onto TPU specimens to track how exposure to the graft material might change the secretory profile and protein expression of these cells. Most adipokines remained unchanged in the secretomes, with the secretion of the hepatocyte growth factor (HGF) being the only substantially reduced (Fig. 4A). The normalization of adipokine levels to the control group (TCP) identified 14 adipokines being substantially up-regulated in the lysates of cells seeded directly onto TPU (Fig. 4B). To explore potential functional relationships among these up-regulated adipokines, a STRING network analysis was performed. The network, shown in Fig. 4C, revealed several highly interconnected adipokines, including insulin-like growth factor (IGF) binding proteins 1 and 2 (IGFBP1 and IGFBP2), which exhibited strong connectivity and the highest up-regulation (log_2_FC ≈ 3.1). Dipeptidylpeptidase 4 (DPPIV) and CC-chemokine ligand 5 (CCL5) also displayed notable up-regulation, while their associated cytokines interleukin-10 (IL-10) and IL-6 showed weaker induction (log_2_FC ≈ 1.6). Interleukin-11 (IL-11) and leptin were also shown to be strongly connected to these 2 cytokines but showed moderate up-regulation (log_2_FC ≈ 2.5 to 3). Interestingly, despite the substantial elevation of angiopoietin-like 3 and receptor for advanced glycation end products in TPU-seeded cells, these factors appeared as isolated nodes in the network, suggesting limited interaction with other regulated adipokines. When the interaction threshold was increased to 0.9, 2 primary clusters emerged (Fig. 4D): one comprising proteins involved in inflammatory regulation (leptin, IL-6, IL-10, IL-11, and CCL5) and another enriched in growth signaling molecules (IGF1, IGF2, IGFBP1, and IGFBP2). GO overrepresentation analysis further revealed T cell and lymphocyte regulation as well as cell–cell adhesion control as the primary biological processes in which the detected proteins are involved (Fig. 4E).

### Biodegradable grafts show early endothelial coverage and vascularized neotissue formation

**Fig. 5.**
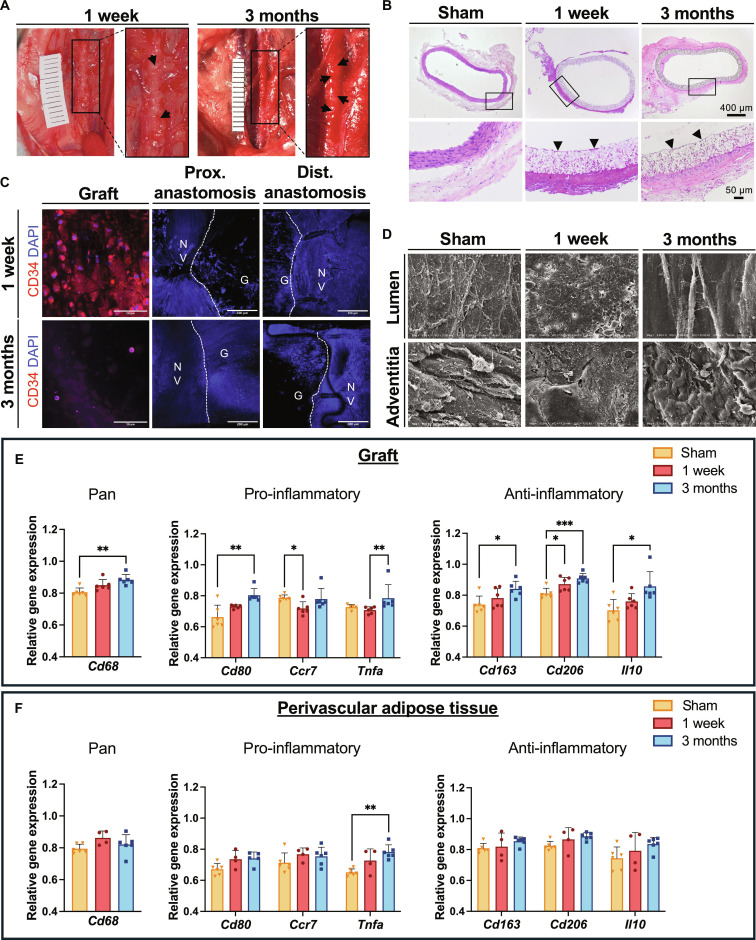
In vivo implantation. (A) Prostheses 1 week and 3 months postimplantation. Arrows indicate small vessel formation within the tissue. (B) Hematoxylin and eosin (H&E) staining of graft explant sections. Arrows show endothelial cell attachment and layer formation on the luminal graft side. (C) Immunofluorescence staining of the graft midsection as well as anastomoses. Red = CD34; blue = nuclei. NV, native vessel; G, graft. (D) Scanning electron microscopy (SEM) images of the graft lumen and adventitia at explantation timepoints. (E) Gene expression of pro- and anti-inflammatory markers in the thermoplastic polyurethane (TPU) graft and (F) in the adjacent perivascular adipose tissue (PVAT). Expression is displayed relative to the expression of Gapdh as a housekeeping gene and presented as mean ± SD. ***P ≤ 0.001; **P ≤ 0.01; *P ≤ 0.05.

The in vivo performance of degradable TPU vascular grafts was evaluated in a small animal model. No signs of thrombus formation were observed at either timepoint, and blood analyses revealed no evidence of systemic inflammation (Fig. S3B to E). By 1 week postimplantation, highly vascularized tissue had formed around grafts and further expanded until 3 months (Fig. 5A, indicated by arrows). Hematoxylin and eosin staining of graft sections confirmed neotissue formation and demonstrated successful luminal adhesion of ECs within 7 d (Fig. 5B). The electron microscopy images of explanted grafts confirmed the attachment of cells at day 7 and an intact endothelium on the luminal side after 3 months, along with cell attachment on the adventitial surface (Fig. 5D). Figure 5C shows transanastomotic ingrowth of ECs from the proximal and distal anastomoses already after 1 week. Three months after implantation, a more confluent layer was observed at the proximal anastomosis compared to that at the distal anastomosis. Additionally, CD34^+^ endothelial progenitor cells were detected on the luminal surface of the graft after 1 week but were scarce at 3 months postimplantation.

### TPU induces distinct inflammatory signatures in vascular and perivascular tissues

To assess the local inflammatory response to the graft material, the expression levels of pro- and anti-inflammatory genes were quantified by performing qPCR on explanted samples. The graft, adjacent PVAT, and both anastomotic regions were examined to distinguish regional cellular responses within the TPU graft and surrounding tissue.

Within the graft, the pan macrophage marker *Cd68* showed a continuous increase, reaching substantially higher levels at 3 months compared to the sham (Fig. 5E). In contrast, CD68^+^ macrophages in the adjacent PVAT showed trends of increasing levels upon implantation, which declined by 3 months postsurgery (Fig. 5F). While in the PVAT, *Tnfa* was up-regulated within the first week and increased further until reaching substantial high levels at 3 months; the pro-inflammatory marker remained unchanged during the first week in the graft wall but became substantially elevated between 1 week and 3 months. Cd80 and *Ccr7* regulation also diverged between the 2 compartments: Whereas *Cd80* was elevated in both compartments 1 week postsurgery, substantial up-regulation at 3 months was detected only in the graft wall. In the graft, *Ccr7* decreased markedly until 1 week postimplantation before rising again until 3 months, whereas in the adjacent PVAT, no substantial changes were observed.

Anti-inflammatory markers *Cd163*, *Cd206*, and *Il10* were all substantially up-regulated in graft samples at 3 months postsurgery (Fig. 5E). Interestingly, these markers showed trends of rising levels but remained largely unchanged throughout the observation period in the PVAT (Fig. 5F). Assessing *Jagged1* expression in graft-surrounding PVATs additionally showed a substantial up-regulation from 1 week to 3 months (Fig. S4A).

Comparative analysis of both anastomotic regions revealed site-specific expression dynamics.

While nearly all analyzed genes—especially pro-inflammatory—were substantially up-regulated over time at the proximal anastomosis, no substantial expression changes were detected at the distal anastomosis (Fig. S4B and C). Notably, *Il10* was the only anti-inflammatory marker substantially increased at the proximal site.

### TPU-associated PVAT shows distinct immune cell activation and SMC plasticity

**Fig. 6.**
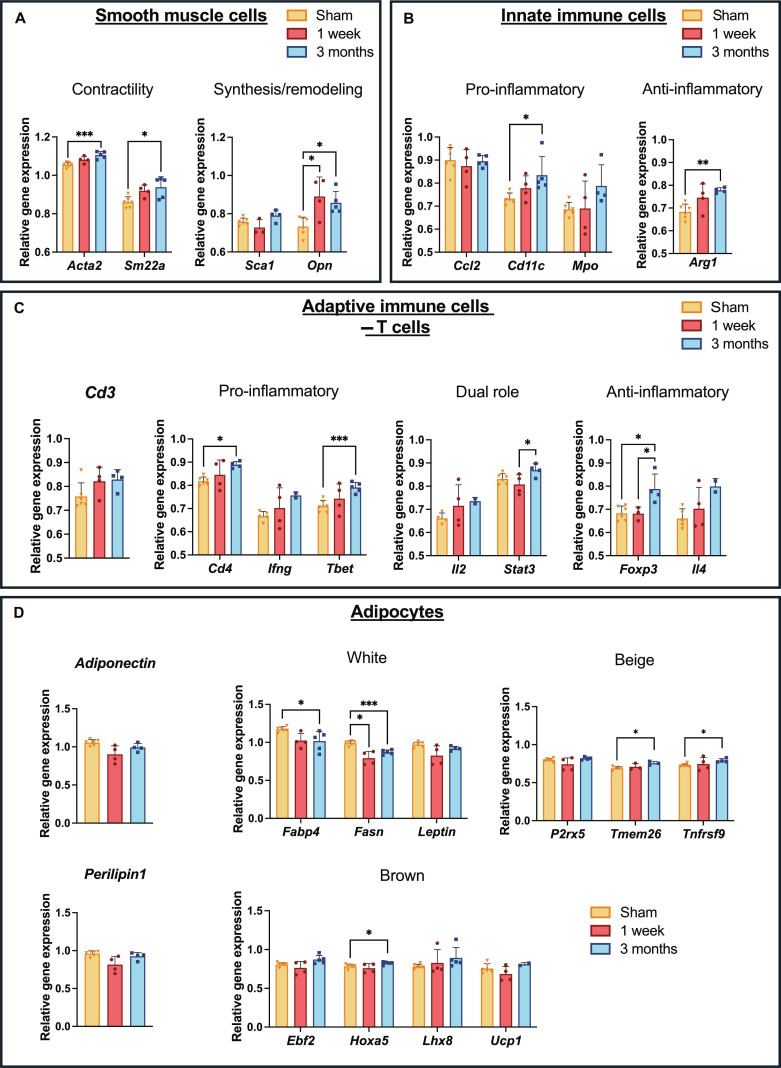
Gene expression profiles within perivascular adipose tissue (PVAT). (A) smooth muscle cell (SMC) markers, (B) innate and (C) adaptive immune cell markers, and (D) adipocyte-subtype-specific markers. Gene expression is displayed relative to the expression of Gapdh as a housekeeping gene and presented as mean ± SD. ***P ≤ 0.001; **P ≤ 0.01; *P ≤ 0.05.

In addition to the previously described markers, the expression of SMC and immune cell markers was analyzed in the TPU-surrounding PVAT. SMC markers associated with the contractile phenotype showed continuous expression elevation with substantially higher levels at 3 months compared to the sham (Fig. 6A). Interestingly, *osteopontin* (*Opn*), indicative of the synthetic and migratory SMC phenotype, was substantially elevated in TPU-PVAT samples after 1 week compared to that in sham controls. Although the expression declined by 3 months postsurgery, it remained substantially above sham group levels. Stem cell marker *Sca1* expression showed no substantial changes over time. Regarding the innate immune response, substantial differences between sham samples and 3-month samples were detected for the pro-inflammatory marker *Cd11c* (Fig. 6B). However, the anti-inflammatory macrophage marker *Arg1* displayed a similar pattern of substantially increased expression. Given the 3-month implantation period, adaptive immune cell markers were assessed to gain insight into long-term host reaction to the biomaterial and revealed an increased yet not substantial elevated presence of T cells at 3 months. Although all T cell markers showed an upward trend over time, only a few showed statistically substantial differences between the groups (Fig. 6C). *Tbet*, a marker of T helper 1 (Th1) polarization, and *Cd4* were substantially elevated after 3 months. The gene expression of signal transducer and activator of transcription 3 (STAT3), which is described as a regulator in both pro- and anti-inflammatory processes, was substantially up-regulated in TPU-PVATs between 1 week and 3 months. However, both anti-inflammatory T cell markers showed an increase in expression over time as well, with *Foxp3* demonstrating highly substantial up-regulation in PVAT at 3 months postimplantation.

### Graft implantation drives phenotype shift from white to brown adipocytes

Figure 6D shows the expression levels of various markers associated with white, beige, and brown adipocyte phenotypes. *Adiponectin* showed slightly decreased expression levels at 1 week, which were normalized to sham levels after 3 months postimplantation, yet not substantial. White adipocyte markers showed decreased levels at the 1-week timepoint with substantial reduction in *fatty acid synthase binding protein 4* (*Fabp4*) and *fatty acid synthase* (*Fasn*). Although expression levels were partly restored to sham levels by 3 months, this was not observed for *Fabp4*, which in contrast decreased further over the study period. Interestingly, beige-adipocyte-related markers substantially increased until 3 months, accompanied by the brown adipocyte marker *homeobox A5* (*Hoxa5*).

## Discussion

This study aimed to evaluate the healing process of biodegradable TPU vascular prostheses, focusing on the formation and plasticity of PVAT surrounding the implanted grafts. The results of this study demonstrated that TPU grafts support the attachment and viability of vascular and perivascular cell types while modulating tissue composition in a material-dependent manner.

In vitro, TPU grafts supported EC, SMC, and PVAT-derived cell attachment and viability (Fig. 2A and Fig. S2). TPU maintained EC characteristics and attenuated the inflammatory response, as reflected by stable CD31 expression and decreased ICAM1 and VCAM1 expression on TPU-seeded ECs (Fig. 2C). Since ICAM1 and VCAM1 mediate leukocyte adhesion and infiltration, their decline represents a beneficial reduction in inflammatory activation that contributes to lowering the risk of intimal hyperplasia and subsequent graft failure [29]. Together, these combined effects are favorable for establishing a nonthrombogenic, quiescent endothelium [4]. Given that insufficient endothelialization remains a central challenge in SDVG design [30], these findings support the translational potential of TPU as vascular graft material. PVAT-derived cells cultured on TPU demonstrated elevated expression of adiponectin (Fig. 2B), an adipokine with well-described anti-inflammatory and vasoprotective effects that enhances endothelial NO availability and inhibits SMC proliferation [31]. Although adiponectin also increased in controls, the greater up-regulation in cells on TPU highlights a material-mediated effect. Additionally, SMCs on TPU displayed lower COL1 expression than controls (Fig. 2D), pointing to advantageous TPU properties, as excessive extracellular matrix (ECM) production contributes to the fibrotic encapsulation of biomaterials and luminal narrowing of the vessel [32].

TPU induced a transient EC activation by regulating the genes *Vec*, *Angpt1*, and *Angpt2* that resolves over time (Fig. 3B), further supporting the development of a stable, functional endothelial phenotype. Moreover, a more contractile and less migratory SMC phenotype was promoted, indicated by higher levels of *Acta2*/*Cnn1* in SMCs on TPU than in SMCs on glass (Fig. 3C) [33]. *Tgfb* and *Timp1* were substantially up-regulated in both groups, suggesting the activation of matrix-modulatory and fibrotic pathways, with higher levels detected in TPU-seeded cells [34]. Elevated *Cd68* expression further supports this interpretation, as *Cd68* is associated with cellular plasticity and transition toward a macrophage-like phenotype during pathological vascular remodeling [35]. The enhanced adipogenic profile of TPU-seeded PVAT cells reflects the material’s distinct mechanical properties, as soft matrices were earlier reported to promote adipogenesis, with key genes such as *Fabp4* peaking at low stiffness [36]. Accordingly, TPU, being more flexible and elastic than rigid TCPs, appears to support a more mature adipocyte phenotype. This was underscored by the higher expression of *perilipin1* and elevated *adiponectin*, indicating adipocyte maturation, lipid accumulation, and a shift toward an anti-inflammatory state in response to the graft material. This interpretation is also supported by a recent comparative SDVG study in which adipocytes seeded onto TPU-based grafts showed a higher adiponectin-associated IF signal than cells on the stiffer ePTFE material, consistent with a more favorable perivascular response to the polyurethane-based graft in vivo [22]. Furthermore, *Leptin* was the only white pro-inflammatory marker that substantially increased in TPU-seeded cells after 3 d (Fig. 3A), but expression declined after 7 d of culture. Aligning with earlier observations on matrix stiffness-dependent adipocyte gene regulation [36], unchanged beige and brown markers indicate that TPU selectively reinforces a white adipocyte program without inducing browning in the absence of dedicated stimuli.

As the regulatory function of adipocytes is largely mediated via adipokines, an adipokine array was performed to evaluate alterations in the adipokine secretion profile in response to TPU. Together with previous reports on declined HGF secretion during adipocyte differentiation [37] and the observed elevated *perilipin1* and *adiponectin* expression, the substantial reduction of HGF detected in the supernatants substantiates the notion that adipocytes on TPU rapidly transition toward a more mature phenotype in vitro (Fig. 4A). A substantially broader response was observed in cell lysates (Fig. 4B), implying enhanced synthesis or intracellular retention. STRING analysis revealed a strongly interconnected network of metabolic and inflammatory regulators. The elevated CCL5 observed in PVAT cells exposed to TPU for 5 d likely reflects an inflammatory response to the graft during an acute phase. Although STRING analysis did not identify a direct interaction, leptin and IL-11 clustered near CCL5, suggesting a potential functional relatedness (Fig. 4C and D). Leptin is known to enhance pro-inflammatory cytokine release (e.g., TNFA) from monocytes during acute inflammation [9], while IL-11 is recognized for its role in driving fibrosis [38]. Most of the up-regulated adipokines are involved in lymphocyte proliferation and activation (Fig. 4E), a process that is triggered during the resolution phase of the acute inflammatory response, several days after biomaterial implantation. At the same time, the healing-associated proteins IL-10 and IGF-1 [39] were expressed alongside with IGFBP1 that supports endothelial repair after injury and NO generation [40]. The simultaneous expression of pro- and anti-inflammatory markers after 5 d suggests that TPU supports the transition from an acute inflammatory response to a remodeling phase. GO overrepresentation analysis further showed that many of the up-regulated adipokines participate in pathways involved in signal transduction and response to chemical or endogenous stimuli, including oxygen-containing compounds (Fig. S5).

The in vivo experiments showed favorable hemo- and biocompatibility. Histology and SEM demonstrated beginning endothelial lining in implanted conduits at 1 week and complete endothelialization by 3 months (Fig. 5B and D), while CD34^+^ cells on the luminal surface at 1 week proved the rapid recruitment of endothelial progenitor cells via fallout endothelialization (Fig. 5C). Moreover, highly vascularized peri-graft tissue developed as early as 1 week postimplantation and continued to expand until 3 months, suggesting PVAT and vasa vasorum formation (Fig. 5A). This is a particularly favorable finding in the context of cell-free graft healing, as recent studies indicate that peri-graft microvascular networks actively support outside-in regeneration and can facilitate endothelial progenitor recruitment and luminal endothelialization [41,42]. Accordingly, increased *Acta2* and *Jagged1* expression in TPU-surrounding PVAT (Fig. 6A and Fig. S4A) indicates recruitment of mural cells and SMCs, contributing to vessel maturation and stabilization [43]. Achieving tissue organization comparable to the native vessel structure is an important indicator of successful biomaterial integration by reducing foreign-body reaction, facilitating mechanical stabilization and supporting long-term functionality.

PVAT’s active contribution to healing was signified by in vivo immune profiling using qPCR on both the graft region and adjacent PVAT, revealing compartment-specific immune responses (Fig. 5E and F). Cd68^+^ macrophages progressively accumulated and persisted within the graft wall, with *Cd68* reaching substantially higher levels at 3 months compared to the sham. Along with the late increase in *Cd80* and *Tnfa*, this suggests delayed pro-inflammatory activation. In contrast, Cd68^+^ macrophages increased transiently in the adjacent PVAT after implantation, consistent with previous reports of an early macrophage influx in PVAT surrounding synthetic grafts [22]. This influx plateaued as the perivascular tissue structure became more comparable to the sham and the acute phase resolved. However, *Tnfa* in PVAT was elevated at 1 week and increased further to substantially high levels at 3 months. With *Cd80* and *Ccr7* largely unchanged, this indicates that rising *Tnfa* may relate to the CD3^+^ T cell presence in PVAT peaking at later timepoints, instead of M1 macrophage population expansion. The markedly decreased *Ccr7* levels in the graft at 1 week that increased again by 3 months may reflect a dynamic shift in macrophage phenotype over time. Despite the late rise in M1 macrophages detected in the graft wall, the substantial up-regulation of the M2 macrophage markers *Cd163*, *Cd206*, and *Il10* at 3 months indicated that the graft milieu is not purely pro-inflammatory but rather characterized by a mixed phenotype. These findings are consistent with reported macrophage heterogeneity during tissue repair, where inflammatory and pro-resolving signals can coexist [44]. Particularly, the increased *Arginase 1* (*Arg1*) at 3 months (Fig. 6B), corresponding with a higher abundance of Cd206^+^ alternatively activated macrophages, indicates active M2-mediated healing mechanisms associated with the resolution of inflammation.

In line with the in vitro data showing the induction of lymphocyte activation and proliferation by TPU, an increased T cell presence was observed within the graft-adjacent PVAT in vivo (Fig. 6C). Substantially up-regulated *Foxp3* expression in PVAT at 3 months denoted an accumulation of regulatory T cells (Tregs), which secrete inflammation-restraining factors [45]. Together with a moderate rise in *Il10* (Fig. 5F), this suggests that the local immune milieu was skewed toward resolving inflammation. *Stat3* up-regulation between 1 week and 3 months further underlines active immune modulation. Because STAT3 mediates IL-10-driven M2 polarization, its elevation at the transcriptomic level supports the emergence of a regulatory, pro-resolving axis within PVAT. Concurrently, elevated *Cd4* levels indicate an increased presence of T lymphocytes in the PVAT, while substantially higher *T-box expressed in T cells* (*Tbet*) suggests that many of these cells were polarized toward a Th1 phenotype [46]. As Tbet is considered a master regulator of CD4^+^ Th1 cells, the rise in Tbet^+^ Th1 cells at 3 months (vs. sham) (Fig. 6C) implies a sustained Th1 cell presence, likely contributing to sustained pro-inflammatory stimuli. The delayed rise of Foxp3^+^ Tregs may represent a feedback mechanism counteracting ongoing Th1/macrophage activity.

The up-regulated *Opn* expression within PVAT adjacent to TPU grafts (Fig. 6A) reflects a sustained activation state that integrates inflammatory signaling with ECM remodeling. Acting as both a pro-inflammatory cytokine and a regulator of ECM dynamics, OPN facilitates macrophage migration, EC and SMC proliferation, fibroblast differentiation, and collagen deposition during wound healing [47]. In the context of SDVG research, elevated *Opn* in PVAT may indicate that the graft-surrounding tissue not only is inflamed but also actively participates in outside-in remodeling, reflecting recent work that emphasizes the importance of the perivascular niche for graft integration [42]. In parallel, increased *Acta2* and *Sm22a* levels indicate a delayed phenotypic shift of mesenchymal stem cells toward a myofibroblast- or SMC-like character [48], supporting the observed microvessel formation within the graft-surrounding tissue.

Given the distinct functional roles of adipocyte subtypes in metabolism and paracrine signaling and their impact on vascular homeostasis, the PVAT analysis was extended to phenotypic observations. At 1 week, PVAT exhibited suppressed expression of the white-adipocyte-associated markers *Fabp4* and *Fasn* (Fig. 6D), reflecting a suppression of lipogenic functions and an injury-induced loss of white adipocyte characteristics following TPU implantation [13]. Down-regulation of both markers reflects early stages of inflammation resolution and the previously described concept of PVAT plasticity. This hypothesis was validated by recovered *adiponectin* levels and progressively increasing beige adipocyte markers *transmembrane protein 26* (*Tmem26*) and *tumor necrosis factor receptor superfamily member 9* (*Tnfrsf9*), with substantially high levels at 3 months. The suggested shift toward activation or expansion of thermogenically competent adipocytes was supported by substantial elevation of *Hoxa5* levels, a transcription factor associated with the brown adipocyte subtype [49]. However, the absence of substantial changes in *Ucp1*, a classical marker for mitochondria-rich brown adipocytes, suggests that browning may have begun but had not progressed to a fully thermogenic state. Notably, TPU promoted a predominantly white maturation in isolated PVAT-derived cells in vitro (Fig. 3A), whereas in vivo, the evolving immune cell niche appeared to favor beiging over time. Since a brown adipose signature is linked to improved vascular reactivity and reduced inflammation, our data showed that TPU promotes a shift toward a more metabolically active and protective PVAT environment [19].

## Conclusion

Overall, the findings of our study demonstrate that bioresorbable TPU grafts contribute to a stabilizing microenvironment with reduced inflammatory activation of vascular and perivascular cells, which is highly relevant for avoiding early thrombo-inflammatory failure of small-diameter grafts in patients. Since a balanced response encourages constructive remodeling, the capacity of TPU to modulate inflammatory signaling emerges as a key design criterion that could be deliberately tuned in future off-the-shelf vascular conduits to support long-term patency in the clinical setting. A particularly novel and translatable aspect of this work is the active role of PVAT in shaping graft integration, especially in the early immune response, underscoring that clinical graft design and implantation strategies should aim to preserve or even harness PVAT rather than neglecting it as a passive bystander tissue. The results highlight PVAT as a dynamic immune and metabolic regulator that actively reacts to its environment and contributes to vascular graft integration, indicating that PVAT-driven adipokine dynamics represent an actionable therapeutic and engineering target—through material choice, surface functionalization, or local drug delivery—to optimize next-generation SDVGs for improved long-term patency in humans.

### Limitations

The small sample size for the in vitro tests represents a limitation of statistical power, which is mitigated by the use of pooled donor cell batches. Since the success of SDVGs does not depend only on the cellular response of one cell type, the use of co-cultures and perfused systems would provide an improved setting to study cell–cell interactions resulting from material-driven responses. Although blood vessel formation was observed around in vivo implanted grafts, CD31 staining combined with lectin perfusion will be required in follow-up experiments to substantiate the indicative data of vasa vasorum formation [50]. Furthermore, graft-adjacent PVAT collected from implants was not sorted using fluorescence-activated cell sorting and therefore did not enable an investigation on the single-cell-type level. Future studies will focus more on metabolomics and lipidomic analyses to better understand the mechanisms underlying the modulation of PVAT by TPU.

## Data Availability

The raw data will be provided upon request.

## Supplementary Materials

Supplementary 1 Graphical Abstract Figs. S1 to S5 Tables S1 to S4
