# Supplementary material for: Exploring Perivascular Adipose Tissue Responses to Bioresorbable Thermoplastic Polyurethane Vascular Grafts
Source: Biomater Res. 2026 May 27;30:0372. doi: 10.34133/bmr.0372 (PMC13213075; doi:10.34133/bmr.0372)
Supplement: Supplementary 1 — Graphical Abstract Figs. S1 to S5 Tables S1 to S4 [file bmr.0372.f1.zip › Graphical Abstract.pdf]

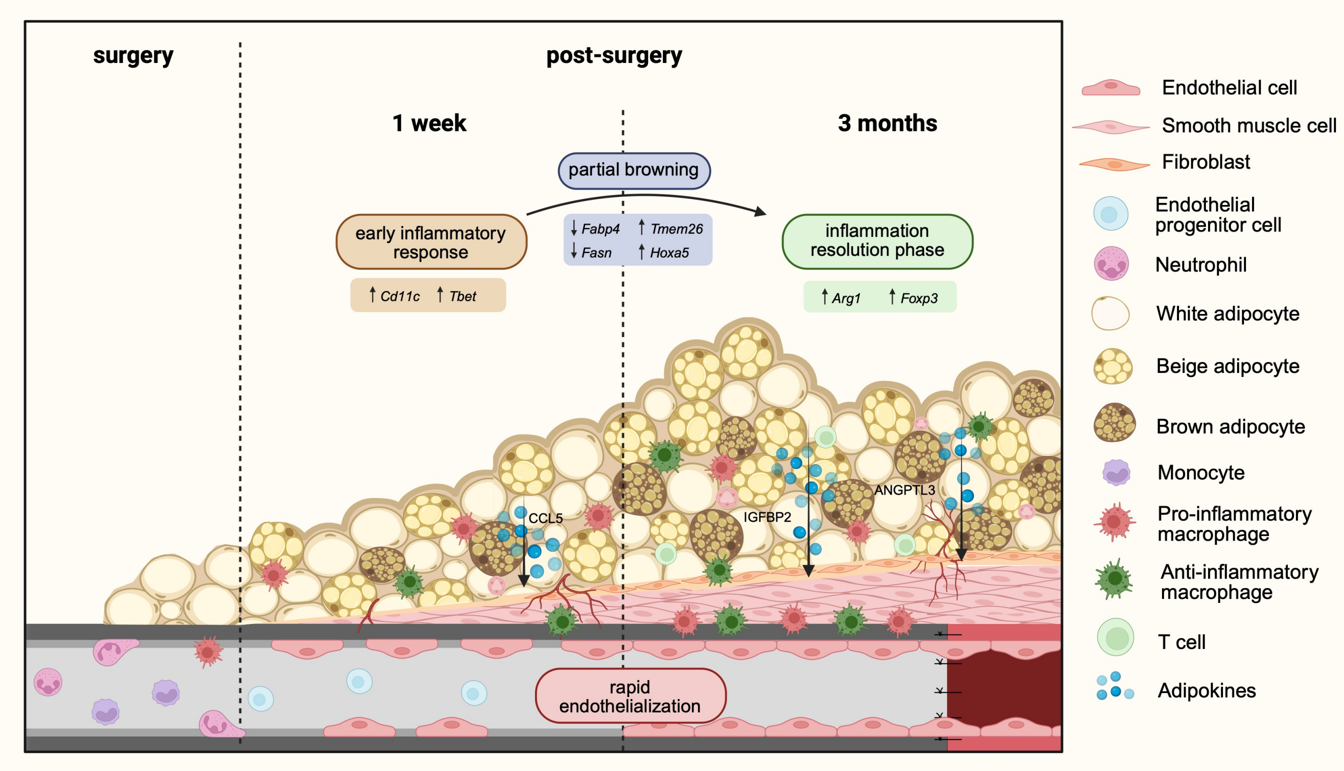

surgery
post-surgery
1 week
3 months
partial browning
early inflammatory response
↑ Cd11c ↑ Tbet
↓ Fabp4 ↑ Tmem26
↓ Fasn ↑ Hoxa5
inflammation resolution phase
↑ Arg1 ↑ Foxp3
CCL5
IGFBP2
ANGPTL3
rapid endothelialization
Endothelial cell
Smooth muscle cell
Fibroblast
Endothelial progenitor cell
Neutrophil
White adipocyte
Beige adipocyte
Brown adipocyte
Monocyte
Pro-inflammatory macrophage
Anti-inflammatory macrophage
T cell
Adipokines
