# Supplementary material for: Exploring Perivascular Adipose Tissue Responses to Bioresorbable Thermoplastic Polyurethane Vascular Grafts
Source: Biomater Res. 2026 May 27;30:0372. doi: 10.34133/bmr.0372 (PMC13213075; doi:10.34133/bmr.0372)
Supplement: Supplementary 1 — Graphical Abstract Figs. S1 to S5 Tables S1 to S4 [file bmr.0372.f1.zip › Supplementary Material Table S1.docx]

**Table S1.** Antibodies and their subsequent dilutions used for *in vitro* IF staining.

| **Antibody** | **Manufacturer** | **RRID** | **Dilution** |
| --- | --- | --- | --- |
| Adiponectin rabbit monoclonal antibody | Abcam, United Kingdom | AB_3105795 | 1:500 |
| CD31 mouse monoclonal antibody | Invitrogen, United States | AB_928130 | 1:100 |
| Collagen 1 mouse monoclonal antibody | Invitrogen, United States | AB_2081889 | 1:500 |
| ICAM1 mouse monoclonal antibody | Santa Cruz, United States | AB_627120 | 1:200 |
| VCAM1 mouse monoclonal antibody | Santa Cruz, United States | AB_626846 | 1:200 |
| Goat Anti-Mouse IgG H&L (Alexa Fluor® 647) polyclonal antibody | Abcam, United Kingdom | AB_2687948 | 1:500 |
| Goat Anti-Rabbit IgG H&L (Alexa Fluor® 488) polyclonal antibody | Invitrogen, United States | AB_143165 | 1:500 |
