# Supplementary material for: Exploring Perivascular Adipose Tissue Responses to Bioresorbable Thermoplastic Polyurethane Vascular Grafts
Source: Biomater Res. 2026 May 27;30:0372. doi: 10.34133/bmr.0372 (PMC13213075; doi:10.34133/bmr.0372)
Supplement: Supplementary 1 — Graphical Abstract Figs. S1 to S5 Tables S1 to S4 [file bmr.0372.f1.zip › Supplementary Material Table S2.docx]

**Table S2.** List of primers used for qPCR.

| **Gene** | **Sequence forward 5‘-3‘** | **Sequence reverse 3‘-5‘** |
| --- | --- | --- |
| *Acta2* | GAGGTATCCTGACCCTGAAGTA | CACACGCAGCTCATTGTAGA |
| *Adipo-nectin* | GCGCTCCTGTTCCTCTTAAT | CATCCAACCTGCACAAGTTTC |
| *Angpt1* | TGGAGAAGCAACTTAGCAGAG | GCAAAGGCTGACAAGGTTATG |
| *Angpt2* | CGGCTGTGATGATCGAGATT | CGAGTCTTGTCGTCTGGTTTA |
| *Arg1* | CCAAGCCAAAGCCCATAGA | CCAGGCCAGCTTTCCTTAAT |
| *Ccl2* | GTCTCAGCCAGATGCAGTTAAT | CTGCTGGTGATTCTCTTGTAGTT |
| *Ccr7* | GACTGAAGACCATGACGGATAC | CACAGGTAGGCACCAAAGAT |
| *Cd11c* | AAGCCCAAGTGTTCCTTCG | AATGACGTGTCGGCTGCT |
| *Cd163* | GCCTCCCAAGAATGACTTTAGA | GGCAATGAGAAGGACCAATAGA |
| *Cd206* | GGGATAGTAAGGCTGCTTGTT | TAGCGGTGTTGAGACTGTAATG |
| *Cd206* | GGGATAGTAAGGCTGCTTGTT | TAGCGGTGTTGAGACTGTAAT |
| *Cd3* | TTCTTCCTTGCTGTTGGTGTAT | GGCTGGTAGACCTGTTCATTT |
| *Cd4* | CACATCCATCACGGCCTATAA | CTCTGCCTTCCATCTCAACTC |
| *Cd68* | CTTGGCTCTCTCATTCCCTTAC | TGTATTCCACTGCCATGTAGTT |
| *Cd80* | CCATCGCCATCATCATCTTCT | GTTTCTCTGCTTGCCTCATTTC |
| *Cnn1* | CAGAGAAACAAGAGCGGAGAT | CTGCTGACTGGCAAACTTATTG |
| *Ebf2* | GCTGCGGGAACCGGAACGAGA | ACACGACCTGGAACCGCCTCA |
| *Fabp4* | CACCTGGAAGAGAACTCCTTG | GAAGCCAACTCCCACTTCTT |
| *Fasn* | GGCGAGTCTATGCCACTATTC | GCTGATACAGAGAACGGATGAG |
| *Foxp3* | ACTGGAGTCTTCTCCCTCAA | TGGGAAGGTGCAGAGTAGA |
| *Gapdh* | ACTCCCATTCTTCCACCTTTG | CCCTGTTGCTGTAGCCATATT |
| *Hif1a* | TCACAAATCAGCACCAAGCAC | AAGGGGAAAGAACAAAACACG |
| *Hoxa5* | CCGACAGAACCAAATCTCTCTAC | CGAGGTTGTAACGTTCCTCTC |
| *Icam1* | GTATCCATCCATCCCACAGAAG | CAGTTGTGTCCACTCGATAGTT |
| *Ifng* | CGAATCGCACCTGATCACTAA | TGGATCTGTGGGTTGTTCAC |
| *Il10* | AGTGGAGCAGGTGAAGAATG | GAGTGTCACGTAGGCTTCTATG |
| *Il2* | GCAGGCCACAGAATTGAAAC | CCAGCGTCTTCCAAGTGAA |
| *Il4* | GTCACCCTGTTCTGCTTTCT | GACCTGGTTCAAAGTGTTGAT |
| *Jagged1* | CTCAGGACCGAACTGTGAAAT | CTGGAGAACACTCACACTCAAA |
| *Klf4* | CAGCTTGCAGCAGTAACAAC | GGATAGCGAGTTGGAAAGGATAA |
| *Leptin* | GGTTTCGTGGTGCTGACTAA | CACATCCTGTTCCGACTCTTAC |
| *Lhx8* | GAGGAGTTTGCCTTGGTAGAG | CCATTACCGTTCTCCACTTCTC |
| *Mmp2* | TGGACTCTAGGAGAAGGACAA | CTGCTGTATTCCCGACCATTA |
| *Mmp9* | CCCAACCTTTACCAGCTACTC | GTCAGAACCGACCCTACAAAG |
| *Mpo* | TGGCTACCCTACTTCCCATAA | CACAGAGAGCACAAGCAACTA |
| *Opn* | AGGAGTTTCCCTGTTTCTGATG | GCAACTGGGATGACCTTGATA |
| *P2rx5* | CTTCAGGTTCGCCAGGTATT | TCCTGCCTTGCCATTAACTATC |
| *Plin1* | GGTGTTACGGATAACGTGGTAG | CTGGAGGATTATCGATGTCTTGG |
| *Sca1* | GGTGTGGCGGTGATACAAT | CCAGCCCTGTCCAAATACAA |
| *Sm22a* | AGAGGACTGTAATGGCTTTGG | CTGTCTGTGAACTCCCTCTTATG |
| *Stat3* | CGCCACTCTGGTGTTTCATA | TTCGCAGGTTGTGCTGATAG |
| *Tbet* | GGAGACACTAAGAGGAGGAGAA | CTGGCCTTCGGTTTCCTTAT |
| *Tgfb* | GCAACAATTCCTGGCGTTAC | GTATTCCGTCTCCTTGGTTCA |
| *Tie2* | CCTTGACAGCACAGGAAGAA | GTAGGTGAGGAGCAGAATTGA |
| *Timp1* | CTAGAGACACGCTAGAGCAGATA | CAGCTACAGGCTTTACTGGAAG |
| *Tmem26* | ATCGGAGGGACCATTACTAGAG | GAGACCAGTGCAGGGTTATTT |
| *Tnfa* | ACCTTATCTACTCCCAGGTTCT | GGCTGACTTTCTCCTGGTATG |
| *Tnfsf9* | GCCTGTGTCCCTATCTTCATC | GTGACCTGGTCTGCAGTATTC |
| *Ucp1* | CCTTTGAGCTCCTCCACAAATA | GGAATCAGCCCTGAAGACAA |
| *Vcam1* | CACCAGACTGTACGATCCTTTC | CTGAACTCCTTGCACTCTACTT |
| *Vec* | CAGTGACAGAGGCCAATACTT | GCCTCCACAGTCAGGTTATAC |
| *Vegfr2* | TCCCAGAGTGGTTGGAAATG | ACTGACAGAGGCGATGAATG |
| *Vimentin* | CCAGGACCTGCTCAATGTAAA | TTGGAAGAGGCAGAGAAATCC |
| *Zic1* | ATATGCGCCAACCCATCAA | TCGTGCATGGTGCTGAAA |
