# Supplementary material for: Exploring Perivascular Adipose Tissue Responses to Bioresorbable Thermoplastic Polyurethane Vascular Grafts
Source: Biomater Res. 2026 May 27;30:0372. doi: 10.34133/bmr.0372 (PMC13213075; doi:10.34133/bmr.0372)
Supplement: Supplementary 1 — Graphical Abstract Figs. S1 to S5 Tables S1 to S4 [file bmr.0372.f1.zip › Supplementary Material Table S3.docx]

**Table S3.** Thermocycler program used for qPCR.

| Hold stage | 50°C for 2 minutes  95°C for 10 minutes |
| --- | --- |
| Amplification (40 cycles) | 5°C for 15 seconds  60°C for 1 minute |
| Melt curve stage | 95°C for 15 seconds  60°C for 1 minute  95°C for 30 seconds  60°C for 15 seconds |
