# Supplementary material for: Exploring Perivascular Adipose Tissue Responses to Bioresorbable Thermoplastic Polyurethane Vascular Grafts
Source: Biomater Res. 2026 May 27;30:0372. doi: 10.34133/bmr.0372 (PMC13213075; doi:10.34133/bmr.0372)
Supplement: Supplementary 1 — Graphical Abstract Figs. S1 to S5 Tables S1 to S4 [file bmr.0372.f1.zip › Supplementary Material Table S4.docx]

**Table S4.** Cobas c311 Analyzer Methods used for hematological analysis.

| **Parameter** | **Method Description** | **Reagent Pack Reference** |
| --- | --- | --- |
| Albumin | Insert.ALB2.0003183688122c501.V11 | 03183688 122 |
| Alkaline Phosphatase acc. IFCC | Insert.ALP2.0003333752190c501.V14 | 03333752 190 |
| Alanine Aminotransferase acc. IFCC | Insert.ALTLP.04467388500.V17 | 04467388 190 |
| Aspartate Aminotransferase acc. IFCC | Insert.ASTLP.0004467493190c501.V18 | 04467493 190 |
| Bilirubin Total | Insert.BILT3.05795397500.V12 | 05795397 190 |
| Cholesterol | Insert.CHOL2.0003039773190c501.V15 | 03039773 190 |
| Glucose HK | Insert.GLUC2.20767131500.V16 | 20767131 322 |
| Total Protein | Insert.TP2.03183734500.V15 | 03183734 190 |
| Triglycerides | Insert.TRIGL.20767107500.V14 | 20767107 322 |
| Urea / BUN | Insert.UREAL.0104460715190c501.V14 | 04460715 190 |
