# Supplementary material for: Exploring Perivascular Adipose Tissue Responses to Bioresorbable Thermoplastic Polyurethane Vascular Grafts
Source: Biomater Res. 2026 May 27;30:0372. doi: 10.34133/bmr.0372 (PMC13213075; doi:10.34133/bmr.0372)
Supplement: Supplementary 1 — Graphical Abstract Figs. S1 to S5 Tables S1 to S4 [file bmr.0372.f1.zip › Supplementary Material.docx]

**SUPPLEMENTARY MATERIALS**

**Fig. S1.** Structural and mechanical characteristics of electrospun TPU grafts. (A) SEM images of cross section (scale bar =200 µm) and luminal and adventitial side (scale bar = 4 µm). (B) Fiber diameter in graft lumen (2.58 ± 0.85 µm) and adventitia (2.25 ± 0.84 µm). (C) Compliance (4.75 ± 0.5 %/100 mmHg) and (D) Tensile force (1.49 ± 0.12 N) of grafts. (E) Suture retention (118.25 ± 18 gram-force) and (F) Gravimetric porosity (52.40 ± 2.3 %). All data were presented as mean ± SD and compared to mechanical properties of the infrarenal rat aorta published by Bergmeister *et al*. [24].

**Fig. S2.** Cell viability assessment of different vascular and perivascular cell types seeded onto TPU grafts *in vitro* for 3 days. PVAT = PVAT-derived cells. EC = Rat aortic endothelial cells. SMC = Rat aortic smooth muscle cells. All data were presented as mean ± SD.

**Fig. S3.** *In vivo* implantation of tubular TPU grafts. (A) Grafts were implanted into the infrarenal abdominal aorta of male Sprague Dawley rats (B) Macroscopic investigation of grafts at experimental timepoints (1 week, 3 months). (C-E) Hematological analysis of sham and operated animals. All data were presented as mean ± SD. ***: *P* ≤ 0.001, **: *P* ≤ 0.01, *: *P* ≤ 0.05.

**Fig. S4.** *In vivo* gene expression (A) *Jagged1* expression in graft-adjacent PVAT. (B-C) Gene expression profile of proximal and distal graft anastomoses. All data were presented as mean ± SD. ***:*P* ≤ 0.001, **: *P* ≤ 0.01, *: *P* ≤ 0.05.

**Fig. S5.** GO overrepresentation analysis of adipokines that were detected being upregulated in PVAT-cells on TPU by performing an adipokine array.
